# Machine Learning and Statistical Analyses of Sensor Data Reveal Variability Between Repeated Trials in Parkinson’s Disease Mobility Assessments

**DOI:** 10.3390/s24248096

**Published:** 2024-12-19

**Authors:** Rana M. Khalil, Lisa M. Shulman, Ann L. Gruber-Baldini, Sunita Shakya, Jeffrey M. Hausdorff, Rainer von Coelln, Michael P. Cummings

**Affiliations:** 1 Center for Bioinformatics and Computational Biology, University of Maryland, College Park, MD 20742, USA; rmkhalil@umd.edu; 2 Department of Neurology, University of Maryland School of Medicine, Baltimore, MD 21201, USA; lshulman@som.umaryland.edu; 3 Department of Epidemiology and Public Health, University of Maryland School of Medicine, Baltimore, MD 21201, USA; abaldin@som.umaryland.edu (A.L.G.-B.); sshakya@som.umaryland.edu (S.S.); 4 Center for the Study of Movement, Cognition, and Mobility, Neurological Institute, Tel Aviv Medical Center, Tel Aviv 6492416, Israel; jeffh@tlvmc.gov.il; 5 Department of Physical Therapy, Faculty of Medical & Health Sciences, Tel Aviv University, Tel Aviv 6997801, Israel; 6 Sagol School of Neuroscience, Tel Aviv University, Tel Aviv 6997801, Israel; 7 Rush Alzheimer’s Disease Center, Rush University Medical Center, Chicago, IL 60612, USA; 8 Department of Orthopedic Surgery, Rush University Medical Center, Chicago, IL 60612, USA

**Keywords:** Parkinson’s disease, mobility tasks, wearable sensors, test–retest reliability, intraclass correlation coefficient, machine learning

## Abstract

Mobility tasks like the Timed Up and Go test (TUG), cognitive TUG (cogTUG), and walking with turns provide insights into the impact of Parkinson’s disease (PD) on motor control, balance, and cognitive function. We assess the test–retest reliability of these tasks in 262 PD participants and 50 controls by evaluating machine learning models based on wearable-sensor-derived measures and statistical metrics. This evaluation examines total duration, subtask duration, and other quantitative measures across two trials. We show that the diagnostic accuracy for distinguishing PD from controls decreases by a mean of 1.8% between the first and the second trial, suggesting that task repetition may not be necessary for accurate diagnosis. Although the total duration remains relatively consistent between trials (intraclass correlation coefficient (ICC) = 0.62 to 0.95), greater variability is seen in subtask duration and sensor-derived measures, reflected in machine learning performance and statistical differences. Our findings also show that this variability differs not only between controls and PD participants but also among groups with varying levels of PD severity, indicating the need to consider population characteristics. Relying solely on total task duration and conventional statistical metrics to gauge the reliability of mobility tasks may fail to reveal nuanced variations in movement.

## 1. Introduction

Parkinson’s disease (PD), a progressive neurodegenerative disorder, poses significant challenges to individuals, affecting their mobility and daily function [1,2,3,4]. Mobility tasks, such as the Timed Up and Go test (TUG), cognitive TUG (cogTUG), and walking with turning (e.g., 32-foot walk with 3 turns), offer valuable insights into the impact of PD on dynamic motor control, balance, and cognitive functions and serve as indicators of an individual’s ability to navigate daily life [5,6,7].

Progress in the field of wearable sensors has enabled the collection of quantitative data from accelerometer and gyroscope recordings. Beyond simply measuring the time taken to perform an entire mobility task, these data can be leveraged to calculate the duration of each subtask of the test, along with numerous measures that provide a detailed characterization of movement. Evidence that this type of granular movement analysis of mobility tasks provides added value over simply timing the overall duration with a stopwatch has been accumulating in recent years. For example, a study highlighted significant differences in TUG-derived acceleration measures between PD participants and age-matched controls, but most measures did not show a correlation with the total duration of TUG [8]. When comparing older adults experiencing falls of unknown origin with healthy controls, the same group of researchers showed significant differences in TUG-derived acceleration measures, but not in overall TUG duration based on a stopwatch [9]. These results suggest that granular sensor-derived TUG measures provide clinically relevant data beyond those captured by overall duration. Similarly, five different sensor-derived TUG subtask measures were associated with clinical parkinsonian gait features in a large community-based cohort study of aging [10], which established the protocol of instrumented mobility tests that we replicated in our study presented here. In a separate study, the use of a single short-duration test and a smartphone-embedded inertial measurement unit showed that individuals with mild to moderate PD exhibit impaired postural control, an altered gait strategy, and slower turn-to-sit performance compared to healthy individuals of the same age [11].

Evaluating the reliability of mobility tasks is essential to ensuring robust and consistent measurements in clinical and research settings [12,13,14,15]. In essence, reliability measurements play a crucial role in assessing the degree to which clinical test scores are affected by measurement errors [16]. A high level of reliability suggests consistent results on repeated assessments, often quantified by the intraclass correlation coefficient (ICC) [17]. A higher ICC value indicates better measurement consistency.

Although previous studies have examined the reliability of certain motor tasks, they often had limitations such as small sample sizes [12,13,18,19,20,21,22,23], a focus on a single population [13,19,20,21,23,24,25,26], or testing only the reliability of the total time taken to complete the task [12,25,26]. Furthermore, all reviewed studies relied solely on statistical measures for the reliability assessment [12,13,18,19,20,21,22,23,24,25,26,27]. However, machine learning has increasingly been applied to the diagnosis of PD using sensor data collected during movement tasks. Thus, evaluating the predictive performance of different trials using machine learning provides a purpose-specific (i.e., diagnostic) evaluation that transcends standard statistical evaluation of test–retest reliability. ML is known for its ability to capture complex patterns and relationships that may be challenging to detect using standard observational and statistical methods [28]. Whereas some research has shown differences in time and sensor-derived measures across disease-defined subpopulations [12,18,22,27], they did not investigate how test–retest reliability varies among these disease severity groups.

Here, we present the results of a comprehensive analysis of test–retest reliability for TUG, cogTUG, and walking with turns, employing two trials for each of these three tasks in PD participants and controls. We compare the performance of machine learning models constructed with quantitative sensor-derived features obtained from subtasks and selected independently from each trial. In addition, we compare the total duration, duration of subtasks, and various quantitative measures across both trials and different groups of participants using statistical measures. Our goal is to address the limitations of previous studies by including a large cohort of 262 participants with PD and 50 controls. We aim to go beyond total task duration, investigating the reliability of mobility tasks using more movement-sensitive measures associated with PD. By incorporating both machine learning techniques and statistical methods to assess reliability, we also examine how reliability differs between PD and control groups, as well as within PD subgroups stratified by disease severity.

The study results show that performance differs when features from each trial are analyzed independently by ML models, suggesting non-equivalence of the two trials. Comparison of the accuracy of models based on the first and second trials of each task revealed that repetition of mobility tasks may not be helpful for the diagnostic accuracy, and thus mobility test protocols may be simplified. Statistical analysis also indicates that the duration of the subtasks and the quantitative measures are less consistent between the two trials compared to the total time, which remains more stable. This implies that relying on total duration as the singular metric for assessing reliability may not be sufficient, as it obscures (or rather fails to reveal) differences in movement characteristics detectable with sensor-derived measures. Furthermore, our study demonstrates that the variability between the two trials differs not only between controls and individuals with PD, but also between groups with increasing severity of PD. These findings prompt a reconsideration of how we conceptualize and define a reliable mobility task, emphasizing metrics that better capture changes in movement across different trials.

## 2. Materials and Methods

### 2.1. Participants

The cohort in our study included 50 control participants and 318 individuals diagnosed with parkinsonism. All participants were enrolled in a study on mobility analysis in individuals with parkinsonism at the University of Maryland Movement and Memory Disorders Center (UM-MMDC) during the period from October 2015 to March 2020. All parkinsonism participants were also part of the Health Outcomes Measurement (HOME) study at UM-MMDC, a naturalistic cohort study collecting patient and clinician-reported data on motor symptoms, activities of daily living, and quality of life during routine office visits. Exclusion criteria for the mobility analysis included an inability to stand or walk without assistance (Hoehn and Yahr, H&Y = 5), other conditions unrelated to parkinsonism significantly impacting gait and balance, or failure to provide informed consent during the evaluation.

Of the 318 parkinsonism participants, 293 were diagnosed with idiopathic PD according to the UK Parkinson’s Disease Society Brain Bank Clinical Diagnostic Criteria [29] by a movement disorder specialist at UM-MMDC. The remaining individuals were diagnosed with other forms of parkinsonism and excluded from the data analysis for this study. One participant with PD was unable to complete the study procedures and was subsequently excluded, and thirty others were excluded due to missing data. In this study, 87% of the participating PD patients reported being in a medication ON state. Patients who had previously undergone deep brain stimulation (DBS) surgery were evaluated while in the ON stimulation state. All participants, including controls, provided their informed consent before any study procedures were performed. The study protocols for both the HOME study and the mobility analysis in the parkinsonism study received approval from the University of Maryland Institutional Review Board.

### 2.2. Mobility Assessments

We followed a previously established protocol for instrumented mobility testing [10], commonly used in clinical practice due to the successful application of mobility data in the research of parkinsonism. Participants performed three mobility tasks from that protocol: 1. 32-foot walk, where participants walked a distance of 8 feet (2.4 m), making two round trips without pauses, which involved a total of three turns; 2. two trials of Timed Up and Go (TUG), where participants started by rising from a seated position in a chair, proceeded to walk eight feet to a designated line on the floor, executed a turn, returned to the chair, made a second turn, and finally seated themselves again; and 3. two trials of cognitive Timed Up and Go (cogTUG), which required participants to perform the TUG while concurrently counting backward from 100 in steps of 3. Participants performed the counting backwards in steps of 3 once in a sitting position as a test run immediately prior to the first cogTUG trial to confirm that they understood the cognitive task and were able to perform it. The second TUG and cogTUG trials for each participant took place in less than one minute after their completion of the first trial. The protocol employed in the second trial was identical to the procedures of the initial trial.

### 2.3. Device and Data Acquisition

Data were acquired using a small (106.6 × 58 × 11.5 mm) lightweight (55 g) sensor device (Dynaport MT, McRoberts B.V., The Netherlands, technical specifications: https://www.mcroberts.nl/products/movetest/, accessed on 17 June 2024) placed on the participant’s lower back using a neoprene waist belt. This device is equipped with a triaxial accelerometer (range: ±8 g, resolution: 1 mg) and a triaxial gyroscope (range: ±2000 dps, resolution: 70 dps), capturing activity data at a sampling frequency of 100 Hz. It records the data in three acceleration axes, including the vertical, mediolateral, and anteroposterior directions, and in three angular velocity axes: yaw (rotation around the vertical axis), pitch (rotation around the mediolateral axis), and roll (rotation around the anteroposterior axis). The sensor recordings were started and stopped remotely via wireless Bluetooth signals from a laptop using a handheld device. Digital markers were strategically placed to mark the initiation and completion of each mobility task, facilitating post hoc segmentation. Sensor readings were then transferred to an encrypted notebook computer and the raw data files were moved to a secure data server.

### 2.4. Data Retrieval

We used a custom Matlab (version 9.1) Runtime-based graphical user interface (GUI) to visualize the complete set of accelerometry data. The identification of the 32-foot walk, TUG, and cogTUG tasks was based on the characteristic accelerometry patterns associated with each task, coupled with digital markers recorded during mobility testing. The raw accelerometry data segmented for each specific mobility task were individually saved as a distinct file for subsequent data processing.

### 2.5. Segmentation

For the TUG and cogTUG tasks, we performed segmentation into subtasks and the extraction of mobility measures as previously described [8,9,30]. In brief, specific patterns and peaks in the accelerometry graphs were identified to delineate the stand-to-sit, walk 1, turn, walk 2, and stand-to-sit subtasks for each TUG and cogTUG mobility task, using a second custom-made Matlab (version 9.1) Runtime-based GUI.

The 32-foot walk mobility task was divided into 2 × 16-foot trials by omitting the second turn. The first trial included the first walk, the first turn, and the second walk, while the second trial consisted of the third walk, the third turn, and the fourth walk. Hence, we refer to each trial as a 16-foot walk. Each trial in the 16-foot walk task was further divided into two walking segments and one turn. To identify the turning segment, we used a previously established algorithm [31], which involved calculating the angular position from the angular velocity along the vertical axis using the trapezoidal integration method [32]. The sections of the signals before and after the turn marked the two walking parts.

### 2.6. Quantitative Measures

For each mobility task, we assessed the total duration calculated from sensor readings as one measure. Additional quantitative measures derived from TUG and cogTUG comprise those derived from walking subtasks, such as total walking duration, step count, step duration, and step and stride regularity [30], and frequency domain measures calculated from the power spectral density (PSD) of the signals, including the amplitude, width, and slope of the PSD peak [33]. For the sit-to-stand and stand-to-sit components, we computed measures including duration, range, jerk, standard deviation, and median measures [9]. The turning measures involved the calculation of the duration, step count, amplitude [30], and dominant frequency [34]. In total, 77 measures were calculated for each TUG and cogTUG trial, providing a comprehensive assessment of various aspects of performance.

For the 16-foot walking task, 36 measures were derived. The measures of the walking segments included walk duration, step count, step duration, stride duration, step regularity, stride regularity, and step symmetry. We used the number of peaks detected in the vertical and anteriorposterior acceleration signals independently to represent the step count, with the step duration and stride duration calculated as the mean time between individual steps and strides, respectively. Step regularity was determined by the amplitude of the first peak in the acceleration autocorrelation signal, and stride regularity corresponded to the amplitude of the second peak in the same signal, as defined in a prior study [35]. Step symmetry was defined as the degree of proximity between the ratio of step regularity to stride regularity and the value 1.0 [35]. For the turning phase of the task, we calculated the time taken to complete the turn, the dominant frequency, and the step count. The dominant frequency was defined as the frequency associated with the highest amplitude in the discrete Fourier transform (DFT), which was calculated using the fast Fourier transform algorithm (FFT) [36]. All quantitative measures were computed for both the vertical and anteroposterior directions.

A complete list of all measures along with their descriptions is provided in the Supplementary Table S1.

### 2.7. Feature Selection

To simplify our model while maintaining or improving its performance, we implemented a forward feature selection approach. Our objective was to identify an optimal subset of features that would lead to a more streamlined model. The process began with feature ranking, achieved through a random forest [37] model. The pivotal point in the feature importance plot, known as the elbow point, was determined as the point farthest from a straight line connecting the first and last points on the plot. This elbow point served as the cutoff threshold for selecting a set of candidate features. Multiple random forest models were trained using subsets of the cumulative top-ranked candidate features. The best-performing model was chosen based on its ability to minimize the total Akaike information criterion (AIC) [38], and the corresponding set of features was subsequently used in further analyzes.

### 2.8. Machine Learning Model

We implemented a five-fold cross-validation framework with stratified sampling to generate balanced training and testing splits. This framework was repeated five times, each time using different random seed to produce varied train and test sets, thus improving robustness. Within each fold, the feature selection method detailed in Section 2.7 was applied exclusively to the training data. Selected features from the training set were then applied to the corresponding test set to prevent data leakage and to ensure generalization [39].

Using these selected features, we trained a random forest model with 10K trees on an undersampled version of the training set, matching the minority class size. Each trained model was then used to make predictions on the corresponding test set. This process generated five predictions per participant, one from each cross-validation replicate. The final class for each participant was determined by a majority vote across these independent predictions.

### 2.9. Statistical Analysis

Data analysis was performed with R version 4.2.3 (15 March 2023) and RStudio. Continuous measures were presented as mean ± standard deviation (SD). To assess test–retest reliability, we used the intraclass correlation coefficient (ICC) and computed 95% confidence intervals (CIs). The analysis used a two-way mixed effects model with absolute agreement using the following formula [40].

$$ICC=\frac{MS_{P}-MS_{E}}{MS_{P}+\left(k-1\right)MS_{E}+\frac{k}{n}\left(MS_{T}-MS_{E}\right)}$$

where $MS_{P}$ = mean square for participants; $MS_{E}$ = mean square error; $MS_{T}$ = mean square for trials; *n* = number of participants; and *k* = number of trials. We interpreted ICC values as follows: values less than 0.5 indicate poor reliability, those between 0.5 and 0.75 denote moderate reliability, values between 0.75 and 0.90 indicate good reliability, and values greater than 0.90 indicate excellent reliability [17]. Additionally, we calculated the standard error of measurement (SEM) using the formula $SEM=SD\times \sqrt{1-ICC}$. SEM quantifies the measurement error in the same units as the measurement, with a lower SEM signifying higher reliability [41]. We then calculated the minimal detectable change (MDC) based on the SEM as follows: $MDC=SEM\times 1.96\times \sqrt{2}$. MDC represents the smallest change required to confidently distinguish the real change between two trials from the changes attributed to the measurement error [42]. Moreover, the level of discrepancy between the two trials was visualized using Bland–Altman plots [43]. These plots feature 95% limits of agreement (LoA), defined as the mean difference ± 1.96 × SD. In these plots, the differences between the two trials are plotted against the mean of the trials. Bland–Altman calculations were performed using the blandr R package.

## 3. Results

Wearable sensor data were collected from 262 participants diagnosed with PD and 50 age-matched controls. Within the PD group, participants were categorized based on their H&Y stage into groups with mild (H&Y ≤ 2), moderate (H&Y = 2.5 and 3), and severe (H&Y = 4) PD. A summary of the characteristics of the study cohort is presented in Table 1.

Participants completed the mobility tasks described in Section 2.2, with data collection following the procedures outlined in Section 2.3 and Section 2.4. These tasks were then segmented as detailed in Section 2.5, producing the quantitative measures described in Section 2.6. The variability in the duration of the task and the subtasks, along with quantitative measures, was assessed using statistical (Section 2.9) and machine learning (Section 2.8) approaches.

### 3.1. Reliability of Task/Subtask Duration

**Figure 1:**
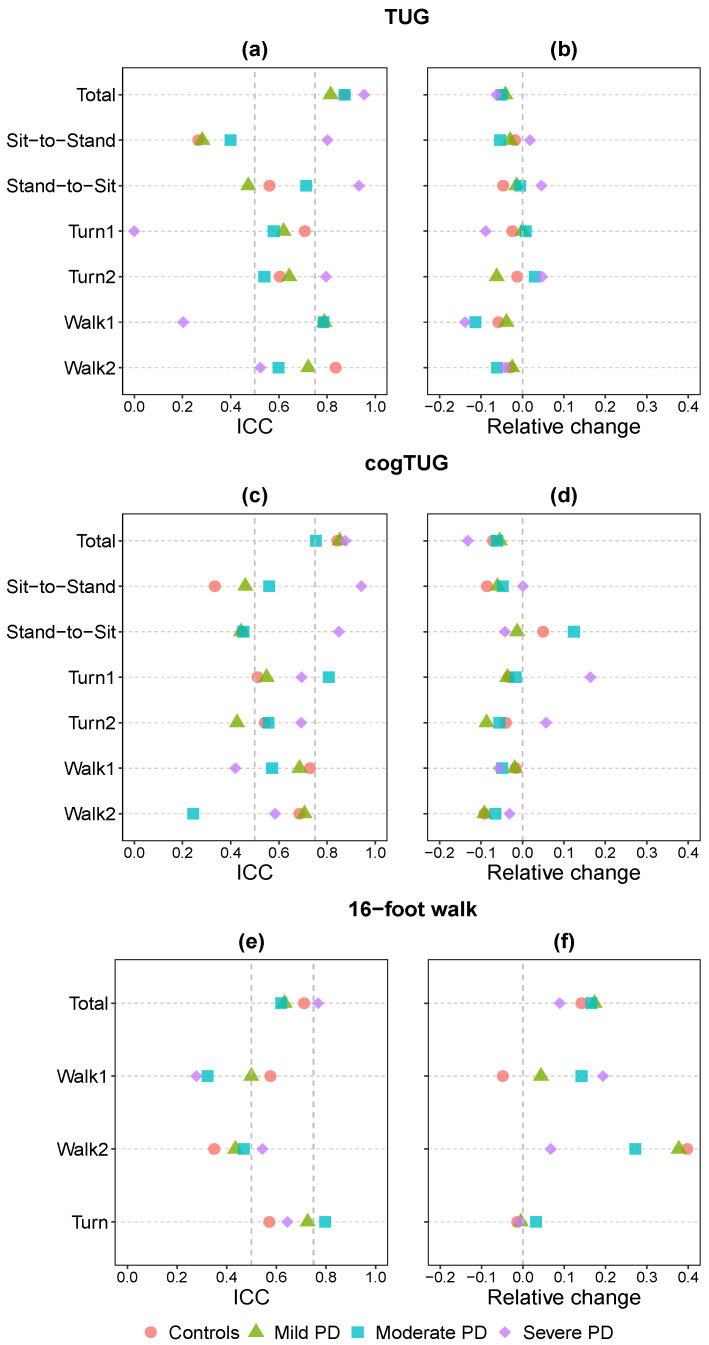
Intraclass correlation coefficient (ICC) (**left**) and median of the relative change (**right**) in total and subtask duration for TUG (**a**,**b**), cogTUG (**c**,**d**), and 16-foot walk (**e**,**f**). Vertical dashed lines represent thresholds for moderate (ICC = 0.5) and good (ICC = 0.75) reliability.

We used sensor readings to measure the time it takes participants to complete the TUG, cogTUG, and 16-foot walk tasks. The ICCs for task duration for each mobility task and participant group are provided in Supplementary Table S2. The reliability of TUG and cogTUG was good to excellent in all groups (ICC = 0.75 to 0.95), and the 16-foot walk task demonstrated moderate to good reliability (ICC = 0.62 to 0.77) across all groups. Participants in the severe PD category showed the highest ICC scores across different tasks (Figure 1a,c,e, top row (“total”)), even though, in general, gait variability increases with progression of PD.

To further characterize the difference between the first and second trials in our mobility tasks, we computed the relative change (RC) in time between the two trials for each task and participant, as another reliability measure (RC = (duration of the second trial − duration of the first trial)/duration of the first trial). Ideally, for a task to be perfectly reliable, the RC of time should be zero. Analysis of the median RC for all participants in each group revealed negative RC values for all groups for TUG and cogTUG, indicating that the duration of the second trial was shorter than the first (see Figure 1b,d, top row). This observation suggests a learning effect in both the TUG and cogTUG tasks for all groups. However, the 16-foot walk showed positive RC values for all groups (Figure 1f, top row), indicating that the second trial took longer than the first, perhaps due to fatigue, as both trials were performed immediately back-to-back (as part of the 32-foot walk mobility task; see Section 2.2).

The fact that the severe PD group exhibited the greatest (negative) RC, despite having the highest ICC values, in both TUG and cogTUG, appears contradictory at first glance. However, mathematically, ICC depends both on differences between participants and between trials (see Section 2.9). Consequently, there is a positive correlation between variance and ICC, with higher variance leading to higher values of ICC. The severe PD group displayed the highest variance among participants by an order of magnitude compared to other groups (see Supplementary Table S3). Thus, even though there is a substantial change in performance between the first and second trials (indicated by a high RC value), the ICC remains high because the numerator of the equation (see Section 2.9) is large. This analysis suggests that, under certain circumstances, depending only on the ICC to measure test–retest reliability can be misleading, especially when testing patients in more advanced stages of PD. It highlights the need to complement the ICC with the RC measure.

In addition to the total duration, we investigated the test–retest reliability of each subtask. Figure 1a,c,e illustrate the difference in ICC values across various subtasks and different groups of participants. Specifically, in the TUG task, all subtask durations exhibited lower ICC values compared to the total duration across all participant groups. Similarly, in the cogTUG task, this trend persisted, except for the severe PD group, where the sit-to-stand subtask displayed a higher ICC value than the total duration. Also, the moderate PD group showed the first turn with the highest ICC score. Furthermore, in the 16-foot walk, the turn subtask exhibited a higher ICC value than the total duration for the mild and moderate PD groups.

Comparing ICC values across different subtasks, we observed that for the TUG task, the walking subtasks had the highest ICC values, followed by the turning subtasks and then the chair transition subtasks (sit-to-stand and stand-to-sit) for both the control and mild PD groups. Walking subtasks also had higher ICC values than turning for the moderate group. However, for severe PD stages, the chair transition subtasks had the highest ICC values. In the cogTUG task, the walking subtasks had the highest ICC values, followed by the turning subtasks, and then the chair transition subtasks for controls, similar to the TUG results. Walking subtasks again had the highest ICC values for the mild PD group and no specific ordering was observed for the moderate PD group. For severe PD stages, the chair transition subtasks had the highest ICC values, similar to the TUG. In the 16-foot walk task, the turning subtasks had higher ICC values than the walking subtasks for all groups of participants.

Figure 1b,d,f shows that possible learning and fatigue effects are not universally associated with all subtasks. For instance, for cogTUG, the severe PD group exhibited a possible learning effect for the overall duration of the task (negative RC for total duration), but both turns had positive RC, indicating that they were slower in the second trial (Figure 1d). Similarly, for the 16-foot walk, controls showed a possible fatigue effect overall (positive RC for total duration), but the first walk component was actually faster in the second trial (negative RC for walk 1; Figure 1f). The absolute values of the RC for the duration of some subtasks were also higher than the absolute RC of the total duration, indicating less consistency between the two trials for those subtasks. On the other hand, the duration of the first turn of TUG, cogTUG, and the 16-foot walk turn showed small RC compared to other subtasks, except for severe PD. In summary, there is a significant degree of variability in test–retest reliability for different subtasks that is not reflected in the high ICC and low RC for the total duration of the task alone.

### 3.2. Reliability of Other Quantitative Measures

Before assessing the test–retest reliability of the sensor-derived quantitative measures, we objectively reduced the number of features, focusing solely on the most important ones. For each task, we constructed three random forest models using all features computed from both trials and aimed to distinguish participants with mild, moderate, and severe PD from the controls. We identified the important features of each model using the feature selection approach described in Section 2.7. The combination of the reduced measure sets from these three models yielded 11 measures for each of the TUG, cogTUG, and 16-foot walk tasks. The consistency of these final sets of selected features between both trials was then evaluated. The mean and standard deviation values for each measurement (feature) are presented in Supplementary Table S4. Supplementary Table S5 includes the ICC, SEM, and MDC values for each measure. Of the 33 quantitative measures, only 1 variable derived from the cogTUG task demonstrated good reliability, with an ICC > 0.77 across all participant groups.

**Figure 2:**
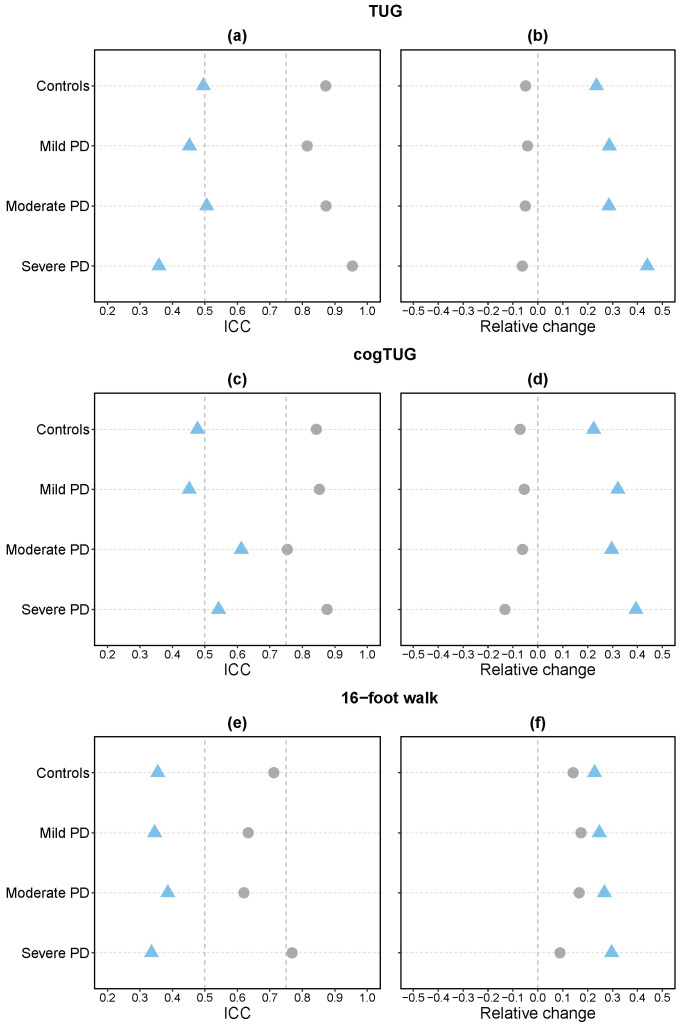
**Left**: Intraclass correlation coefficient (ICC) of total duration and other quantitative measures for TUG (**a**), cogTUG (**c**), and 16-foot walk (**e**). Gray points represent the ICC of the total duration and blue triangles represent the median of the ICC values across the other quantitative measures. Vertical dashed lines represent thresholds for moderate (ICC = 0.5) and good (ICC = 0.75) reliability. Median ICC values, along with their respective confidence intervals, across the quantitative measures for each task and participant group are presented in Supplementary Table S6. **Right**: Relative change (RC) in total duration and other quantitative measures for TUG (**b**), cogTUG (**d**), and 16-foot walk (**f**). Gray points represent the median of the RC of the total duration across participants of each group. Blue triangles represent the median of the medians of the absolute RC values across participants and quantitative measures.

Taking the median, which is a robust statistic, of the ICC values across all measures, the selected quantitative measures exhibited lower scores compared to the total duration of each of the three mobility tasks (Figure 2a,c,e). Although the ICC values for the total duration of both TUG and cogTUG demonstrated good to excellent reliability for all groups of participants, the median ICC values for other measures ranged from poor to moderate reliability (ICC = 0.36 to 0.61). Similarly, although the total duration of the 16-foot walk displayed moderate to good reliability, the median ICC values for other quantitative measures exhibited poor reliability (ICC = 0.34 to 0.39). ICC scores for duration and other measures were also different among the groups of participants. The median ICC values, along with their respective confidence intervals, across the quantitative measures for each task and group of participants are presented in Supplementary Table S6.

We also determined the median of the median values of RC for the quantitative measures by calculating the median of the absolute value of RC across all participants in each group and then determining the overall median from all measures. The RC values for the quantitative measures were higher (indicating less consistency) than the RC values for the total duration of the three tasks (Figure 2b,d,f). The greater change in other mobility measures between trials compared to total duration is consistent with our observation of a much greater variability of subtask duration than the total duration between trials (demonstrated in Figure 1). Among the participant groups, the controls displayed the lowest RC value, and the severe PD group exhibited the highest value (i.e., the greatest amount of change between the first and second trials) in all tasks.

For a more granular analysis, for each mobility task, individual measure-level ICC scores and the median of absolute values of RC across all participants in each group were calculated, and these values are visualized in Supplementary Figures S1–S3. There are substantial variations in reliability (ICC and RC scores) among different groups of participants for many measures. A few measures, such as the TUG amplitude yaw of the second turn (Supplementary Figure S1, first measure) and range of sit-to-stand (Supplementary Figure S1, fifth measure), as well as amplitude-yaw of first turn from the cogTUG task (Supplementary Figure S2, tenth measure), showed small differences between participant groups in both ICC and RC values. Only the amplitude yaw of the first turn of the cogTUG task demonstrated good reliability (ICC > 0.77) across all participant groups (Supplementary Figure S2, tenth measure). The reliability of other measures varied from poor to excellent across the different groups for the three mobility tasks.

To further elucidate the differences between the two trials, Bland–Altman plots were employed, illustrating the relationship between the differences and average values obtained from a measure in both trials. This graphical approach quantifies the agreement and allows for the identification of any potential systematic bias. We plotted the discrepancies in quantitative measures with 95% confidence intervals (CIs) and 95% limits of agreement (LoA) (see Supplementary Figures S4–S6). Points outside the CI of the upper and lower LoA for certain measures indicate divergent values between the two trials.

### 3.3. Performance of Machine Learning Models

For each mobility task, trial, and group of PD participants, we applied the machine learning modeling approach described in Section 2.8 to distinguish the corresponding group of PD participants from controls (i.e., each analysis is a binary classification problem). The performance of these models is detailed in Table 2. Across the three mobility tasks, there were variations in the accuracy of models built using features calculated from each trial separately for classifying different PD groups and controls. As expected, the accuracy in differentiating PD from control participants was much higher for severe PD compared to mild or moderate disease. When comparing the accuracy of the models based on the first vs. second trial of each task, we observed either a similar or a reduced accuracy from the first to the second trial, with two exceptions (16-foot walk for mild PD and TUG for severe PD). Models using features of the second trial alone showed a decrease in accuracy by a mean of 1.8% compared to models using features from the first trial. Similarly, models incorporating features from both trials showed similar or lower accuracy compared to those using only first-trial features, except in three cases (16-foot walk for mild and severe PD, and TUG for severe PD). These models showed only a slight improvement, with a mean increase of 0.52% over models using first-trial features. This suggests that, for the purpose of differentiating PD from controls, repeating mobility tasks in this study generally provides little to no additional benefit.

To demonstrate how sensor-based measures provide valuable information beyond what task duration reveals, we constructed logistic regression models using only the mean total duration from both trials as a predictor. These models showed a decrease of 7.0% in mean accuracy compared to models that used features from the first trial alone (Table 2). Based on these findings, we conclude that sensor-derived measures provide additional value beyond timing the overall task duration.

## 4. Discussion

Our study presents an evaluation of the test–retest reliability of three commonly employed physical performance tasks for the assessment of PD mobility. Through machine learning and statistical analyses, we demonstrate that, although total duration remains consistent across various trials (ICC = 0.62 to 0.95), there is discernible variation in other sensor-derived measures. These results imply that relying solely on total duration, as commonly practiced, does not adequately capture the true changes in movement between trials. Additionally, we demonstrate that the reliability of sensor-derived metrics varies between different populations and even among subgroups within the same population. Therefore, careful attention should be paid to the population being studied.

We assessed the reliability of mobility tasks by employing machine learning models that go beyond traditional reliance on standard statistical measures [12,13,18,19,20,21,22,23,24,25,26,27]. Specifically, we constructed random forest models to distinguish different groups of PD participants from controls using features calculated independently from each trial, as well as features combined from both trials. Across all tasks, we observed a performance difference between models built using the first trial and those built using the second trial (see Table 2). The pattern of similar or reduced accuracy from the first to the second trial, or when using combined trials, for most cases indicates that, for the purpose of differentiating PD from controls, repeating mobility tasks offers little to no added benefit. In other words, our results suggest that mobility testing protocols can be simplified to include a single trial of TUG, cogTUG, and walking with turning, if differentiating PD from controls is the purpose of mobility testing. This simplification will save time and effort for both participants and clinicians, thereby improving the feasibility of using wearable sensors as a diagnostic tool in clinical settings. Whereas our previous work demonstrated that reducing the number of sensors and tasks can simplify mobility assessment [44], here we recommend minimizing the number of trials per task to one single trial.

On the other hand, the performance variation of the ML models between trials underscores that the sensor-derived measures of each trial may possess distinct values. These differences between task trials affect the ability of the models to differentiate between PD groups and controls. Additionally, we demonstrate that models built using only total duration performed worse in all cases compared to models that used sensor-derived measures. This suggests that sensor-derived measures offer enhanced informativeness by providing a finer resolution of movement characteristics compared to total duration alone [9].

With respect to test–retest reliability, our procedure, in which participants completed two trials of each task back to back during the same clinical visit, represents a best-case scenario. This design limits potential variability due to factors such as changes in disease progression or symptoms, day-to-day differences in physical status that can affect movement, and differences in sensor positioning. Thus, this design ensures that participants are in the same disease state for both trials and all other specific characteristics are identical. Despite controlling for these variables, we still observed trial-to-trial variability. We anticipate that altering any of these factors could further increase variability.

Among previous studies that used the ICC of the total task duration to assess the reliability of various mobility tasks, a study evaluating the reliability of the TUG in PD used the time of five TUG trials, which produced high ICC scores ranging from 0.87 to 0.99 [12]. Similar findings were reported in another study that assessed TUG in PD using the elapsed time of two trials (ICC = 0.80) [25]. Several other studies, spanning various populations, have focused on the total duration to test the reliability of different mobility tasks. For instance, in people with chronic stroke, the reliability of dual-task mobility assessments was investigated, demonstrating good reliability (ICC = 0.70 to 0.93) based on walking time across five different walking tasks [27]. Additionally, TUG reliability was assessed in older individuals over three different days, revealing a good reliability (ICC = 0.92) based on the total time required to perform the TUG [26]. Our results align with the literature, revealing good to excellent reliability of the total duration of TUG and cogTUG (ICC = 0.75 to 0.95) and moderate to good reliability for the 16-foot walk (ICC = 0.62 to 0.77). However, the relative change in time between the trials revealed a possible learning effect in TUG and cogTUG and a possible fatigue effect in the 16-foot walk (see Figure 1). The fatigue effect observed in the 16-foot walk arises from participants completing four 8-foot walks without any rest, in contrast to the TUG and cogTUG, where participants had a break between trials, leading to the observed learning effect. The learning effect aligns with the findings of other studies [13,45]. When comparing our results to those of the other studies referenced above, it is also important to consider the different implications of retesting immediately or within minutes (as in our case), when fatigue and learning effects may play a role, versus retesting over several days, when possible fatigue effects would be expected to be negligible.

In addition to the total duration of the tasks, we conducted an examination of the trial differences by exploring the duration variations in the subtasks that make up the three mobility tasks. As illustrated in Figure 1, the consistency among the different components of each task is not uniform, aligning with prior findings [20,23,46]. For example, a study of 28 PD participants demonstrated that the walking and turning phases of the TUG were more reliable than the sit-to-stand and stand-to-sit phases [23]. Similarly, the sit-to-stand and stand-to-sit phases showed the lowest reliability in a study of 30 young adults [19], as well as in another study that assessed the reliability of a modified version of the TUG with 28 participants experiencing balance and gait problems [20]. The latter study attributed the low reliability to two factors: the short duration of the subtask (around three seconds), which increases the measurement error, and the difficulty in defining the exact end of the maneuver [47,48]. Other studies showed that sit-to-stand had the lowest reliability among the components of TUG in healthy adults [46], as well as in patients with mild PD and age-matched controls [22]. The variability in strategies used to perform sit-to-stand, due to its high degrees of freedom, could explain the poor reliability of this metric [49,50]. Consistent with these findings, we observed that the sit-to-stand subtask was the least reliable in the TUG for both the mild and moderate PD groups, as well as for the controls, and in the cogTUG, it was the least reliable subtask for the controls (see Figure 1).

Furthermore, we explored the reliability assessment of a selected subset of quantitative measures derived from sensor data. Figure 2 highlights that these measures exhibit lower consistency than the total duration across all participant groups, with reduced ICC and higher RC scores. These results are in line with previous research that evaluated the reliability of certain gait [13,21,22], turn [21,22], and transition [22] measures. Once again, our results demonstrate that the total duration of the task does not capture the differences in movement revealed by other measures, highlighting the importance of examining individual components for a more comprehensive reliability assessment. Our results presented here offer a much more granular analysis, determining the test–retest reliability of each mobility measure among the groups of participants with different levels of severity of PD. This is in contrast to previous studies referenced above that conducted reliability analyses using data from the entire study population. The observed variation in reliability for each measure among different participant groups, as depicted in Supplementary Figures S1–S3, underscores the need for tailored assessments that account for the inherent heterogeneity within the studied population.

Among the measures analyzed for both TUG and cogTUG, we found that features derived from the turning subtask had better test–retest reliability compared to those from the sit-to-stand and stand-to-sit subtasks across all PD groups, with the exception of TUG features in the moderate PD group. This aligns with a study that reported lower reliability for trunk kinematics during sit-to-stand and stand-to-sit compared to turns in a PD population with a median H&Y score of 3 [23], and another study that identified sit-to-stand features as the least reliable among the TUG components in a population of 12 early-to-moderate PD patients (H&Y = 1 to 2.5) [22]. The effect of adding a cognitive task to the TUG has also been explored, and previous work has shown a decrease in reliability when a cognitive task was introduced in a population of 12 community-dwelling older adults [13]. Similarly, in our study, the median ICC values across all measures were lower for cogTUG than for the TUG task in the control group (see Figure 2).

The finding that the severe PD group exhibited both the highest ICC and the greatest RC between trials (see Figure 1) suggests that this discrepancy may be an artifact of the ICC calculations, influenced by both within-participant and between-participant variance. A similar observation was made in another study, which noted that variability increases with the time taken to complete the TUG and that ICC is significantly impacted by the range of scores used in its calculation [26]. Specifically, ICC tends to be higher when the differences between measurements are small relative to the range of scores across participants. This underscores a potential source of misinterpretation when relying on ICC, as opposed to RC, which more accurately reflects the temporal differences between trials.

The subsets of sensor-derived quantitative measures analyzed in the test–retest reliability assessment (Section 3.2) and the model performance evaluation (Section 3.3) differ because these analyses are distinct and designed to address separate objectives. Both analyses aim to explore differences between the two trials, but their focus and criteria for feature inclusion differ. In the reliability assessment, we prioritized features that exhibit high variability between trials and substantial differences across participant groups, as these features best illustrate the inconsistency of sensor-derived measures within and between groups. This approach emphasizes understanding the reproducibility of the measures and their potential sensitivity to the characteristics of the participants. In contrast, in the model performance evaluation, the selection of features was guided by the accuracy of the classification. Here, we focus on identifying the most important features distinguishing participants with PD from the controls. This required selecting features based on their importance in model diagnostic performance rather than their trial-to-trial variability. By addressing these separate goals, the two analyses complement each other, providing insights into both the stability and predictive utility of sensor-derived features.

We acknowledge several limitations in our study. First, we split the 32-foot walk task into two 16-foot trials due to a lack of separate trials, which could impact the test–retest reliability results. Furthermore, the MDC values reported in this paper may represent underestimates, as our test–retest procedure did not account for additional sources of variability, such as day-to-day fluctuations, disease symptoms, physical status, or sensor repositioning. A more comprehensive evaluation that incorporates these factors would be necessary for a more accurate calculation of MDC. Furthermore, our findings are based on specific mobility tasks (i.e., TUG, cogTUG, and walking with turns), and may not be generalized to other motor or cognitive assessments used in PD. It is also unclear whether our results will apply to other movement assessment contexts, including disorders other than PD, or the evaluation of movement tasks beyond disease diagnostics, such as in sports performance analysis.

## 5. Conclusions

Our findings reveal that repeating mobility tasks may not be helpful for diagnostic accuracy, allowing for the simplification of mobility test protocols; relative change may be superior (or at least offer important complementary information) to ICC when determining test–retest reliability, depending on the disease severity of the population/group in question; and relying exclusively on the overall task duration for reliability assessment is insufficient, as it neglects analytically important differences in aspects of mobility captured by other quantitative measures. Through machine learning and statistical analyses, we demonstrate the nonidentical nature of the different trials when repeating mobility tasks. Specifically, sensor-derived measures exhibit less consistency between the two trials compared to the total duration of the task, which remains more stable. Our findings also reveal that variability between trials differs not only between controls and PD participants, but also among groups with varying levels of severity of PD. These results prompt a reassessment of how we define a reliable mobility task, urging a more comprehensive and nuanced approach to capturing the complexities of task performance and the inherent heterogeneity within the studied population.

## Figures and Tables

**Table 1:** Characteristics of the study cohort.

Feature	Controls (*n* = 50)	PD (*n* = 262)
Age (years, $\bar{x}\pm SD$)	64.1 ± 9.8	66.9 ± 9.3
Gender (%male)	38.0	62.0
Height (cm, $\bar{x}\pm SD$)	168.1 ± 10.9	172.2 ± 10.4
UPDRS (total, $\bar{x}\pm SD$)	–	35.5 ± 17.1
UPDRS (motor-part III, $\bar{x}\pm SD$)	–	22.2 ± 11.8
Disease duration (years, $\bar{x}\pm SD$)	–	7.8 ± 6.5
H&Y ($\bar{x}\pm SD$)	–	2.2 ± 0.62
Stage 1 (n)	–	12
Stage 1.5 (n)	–	4
Stage 2 (n)	–	169
Stage 2.5 (n)	–	35
Stage 3 (n)	–	25
Stage 4 (n)	–	17

**Table 2:** Accuracy (percent) of the models. Trial 1 models used features derived only from the first trial; Trial 2 models used features derived only from the second trial; Trials 1&2 models used features derived from the first trial, the second trial, and the mean of corresponding features from both trials; and duration models used the mean durations from both trials. Numbers in parentheses represent the difference in accuracy between each model and the corresponding model that used only features from the first trial.

		Trial 1	Trial 2	Trials 1&2	Duration
Mild PD vs. controls					
	TUG	75.3	73.6 (−1.7)	74.5 (−0.8)	59.3 (−16.0)
	cogTUG	81.7	76.6 (−5.1)	81.7 (0.0)	57.6 (−24.1)
	16-foot walk	66.0	66.8 (+0.8)	69.8 (+3.8)	62.3 (−3.7)
Moderate PD vs. controls					
	TUG	80.9	80.9 (0.0)	80.0 (−0.9)	80.0 (−0.9)
	cogTUG	82.7	79.1 (−3.6)	82.7 (0.0)	80.9 (−1.8)
	16-foot walk	74.5	70.9 (−3.6)	73.6 (−0.9)	71.5 (−3.0)
Severe PD vs. controls					
	TUG	91.0	94.0 (+3.0)	94.0 (+3.0)	85.2 (−5.8)
	cogTUG	95.5	89.6 (−5.9)	89.6 (−5.9)	88.6 (−6.9)
	16-foot walk	86.6	86.6 (0.0)	91.0 (+4.4)	86.2 (−0.4)

## Data Availability

De-identified sensor-derived measures have been deposited in the Digital Repository at the University of Maryland (DRUM), https://doi.org/10.13016/jgw3-6sga (accessed on 16 December 2024). The code used in this research paper can be accessed publicly at the same link. The provided notebook contains the code employed for conducting the analysis presented in this study. Comprehensive instructions for configuring the required environment and executing the code are outlined in the README file.

## Supplementary Materials

The following supporting information can be downloaded at: https://www.mdpi.com/article/10.3390/s24248096/s1, Figure S1. TUG intraclass correlation coefficient (ICC) (a) and median of the relative change (b) of each measure. Vertical dashed lines represent thresholds for moderate and good reliability; Figure S2. cogTUG intraclass correlation coefficient (ICC) (a) and median of the relative change (b) of each measure. Vertical dashed lines represent thresholds for moderate and good reliability; Figure S3. 16-foot walk intraclass correlation coefficient (ICC) (a) and median of the relative change (b) of each measure. Vertical dashed lines represent thresholds for moderate and good reliability; Figure S4. Bland-Altman plots of each measure for the TUG task. The dashed lines represent the mean of the differences and the 95% limits of agreement (LoA), defined as mean ± 1.96 SD. The blue and red shaded areas are the confidence intervals of the mean and LoA respectively; Figure S5. Bland-Altman plots of each measure for the cogTUG task. The dashed lines represent the mean of the differences and the 95% limits of agreement (LoA), defined as mean ± 1.96 SD. The blue and red shaded areas are the confidence intervals of the mean and LoA respectively; Figure S6. Bland-Altman plots of each measure for the 16-foot walk task. The dashed lines represent the mean of the differences and the 95% limits of agreement (LoA), defined as mean ± 1.96 SD. The blue and red shaded areas are the confidence intervals of the mean and LoA respectively; Table S1. Sensor-derived quantitative parameters. All quantitative measures were computed for both the vertical and anteroposterior directions; Table S2. Intraclass correlation coefficient (ICC, 95% CI) of sensor-based total duration of each mobility task. CI: confidence interval; Table S3. Variance of sensor-based task duration ($s^{2}$); Table S4. Values of assessments measures; Table S5. Reliability of each of TUG, cogTUG, and 16-foot walk assessments. ICC intraclass correlation coefficient, CI confidence interval, SEM standard error of measurement agreement, MDC minimal detectable change; Table S6. Median of intraclass correlation coefficient (median, 95% confidence interval (CI)) across the quantitative measures.
