# Supplementary material for: Machine Learning and Statistical Analyses of Sensor Data Reveal Variability Between Repeated Trials in Parkinson’s Disease Mobility Assessments
Source: Sensors (Basel). 2024 Dec 19;24(24):8096. doi: 10.3390/s24248096 (PMC11678965; doi:10.3390/s24248096)
Supplement: Supplementary file 1 [file sensors-24-08096-s001.zip › sensors-3281087-supplementary.pdf]

Supplementary Material for Machine Learning and Statistical Analyses of Sensor Data Reveal Variability Between Repeated Trials in Parkinson's Disease Mobility Assessments

Rana M. Khalil[1], Lisa M. Shulman[2], Ann L. Gruber-Baldini[3], Sunita Shakya[3], Jeffrey M. Hausdorff[4,5,6,7,8], Rainer von Coelln[2,*], and Michael P. Cummings[1,*]

[1]Center for Bioinformatics and Computational Biology, University of Maryland, College Park, Maryland, USA
[2]Department of Neurology, University of Maryland School of Medicine, Baltimore, Maryland, USA
[3]Department of Epidemiology and Public Health, University of Maryland School of Medicine, Baltimore, Maryland, USA
[4]Center for the Study of Movement, Cognition, and Mobility, Neurological Institute, Tel Aviv Medical Center, Tel-Aviv, Israel
[5]Department of Physical Therapy, Sackler Faculty of Medicine, Tel Aviv University, Tel Aviv, Israel
[6]Sagol School of Neuroscience, Tel Aviv University, Tel Aviv, Israel
[7]Rush Alzheimer's Disease Center, Rush University Medical Center, Chicago, IL, USA
[8]Department of Orthopedic Surgery, Rush University Medical Center, Chicago, IL, USA
[*]Correspondence: rvoncoelln@som.umaryland.edu (R.vC), mcummin1@umd.edu (M.P.C)

Contents

Supplementary Files **12**

Supplementary Tables

S1 Table S1 2
S2 Table S2 3
S3 Table S3 3
S4 Table S4 4
S5 Table S5 5
S6 Table S6 6

Supplementary Figures

S1 Figure S1 7
S2 Figure S2 7
S3 Figure S3 8
S4 Figure S4 9
S5 Figure S5 10
S6 Figure S6 11

Table S1. Sensor-derived quantitative parameters. All quantitative measures were computed for both the vertical and anteroposterior directions.

Task	Subtask	Measure
TUG, cogTUG	overall TUG	Duration [s] -acc
TUG, cogTUG	overall TUG	Duration [s]- marks
TUG, cogTUG	overall TUG	Standard deviation [g]
TUG, cogTUG	overall TUG	Median [g]
TUG, cogTUG	Walking	Duration (walk 1+ walk 2) [s]
TUG, cogTUG	Walking 1,2	Duration [s]
TUG, cogTUG	Walking 1,2	Number of steps
TUG, cogTUG	Walking 1,2	Step regularity [g^2]
TUG, cogTUG	Walking 1,2	Stride regularity [g^2]
TUG, cogTUG	Walking 1,2	Step symmetry
TUG, cogTUG	Walking 1,2	Step duration [s]
TUG, cogTUG	Walking 1,2	Stride duration [s]
TUG, cogTUG	Walking 1,2	Step duration/stride duration
TUG, cogTUG	Walking 1,2	Frequency (7 s) [hz]
TUG, cogTUG	Walking 1,2	Amplitude (7 s) [psd]
TUG, cogTUG	Walking 1,2	Width (7 s) [hz]
TUG, cogTUG	Walking 1,2	Slope (7 s) [psd/hz]
TUG, cogTUG	Walking 1,2	Number of steps
TUG, cogTUG	Walking 1,2	Step regularity [g^2]
TUG, cogTUG	Walking 1,2	Stride regularity [g^2]
TUG, cogTUG	Walking 1,2	Step symmetry
TUG, cogTUG	Walking 1,2	Step duration [s]
TUG, cogTUG	Walking 1,2	Stride duration [s]
TUG, cogTUG	Walking 1,2	Step duration/stride duration
TUG, cogTUG	Walking 1,2	Frequency (7 s) [hz]
TUG, cogTUG	Walking 1,2	Amplitude (7 s) [psd]
TUG, cogTUG	Walking 1,2	Width (7 s) [hz]
TUG, cogTUG	Walking 1,2	Slope (7 s) [psd/hz]
TUG, cogTUG	Sit-to-Stand	Range [g]
TUG, cogTUG	Stand-to-Sit	Range [g]
TUG, cogTUG	Sit-to-Stand	Duration [s]
TUG, cogTUG	Stand-to-Sit	Duration [s]
TUG, cogTUG	Sit-to-Stand	Jerk [g/s]
TUG, cogTUG	Stand-to-Sit	Jerk [g/s]
TUG, cogTUG	Sit-to-Stand	Jerk sit-to-stand A [g/s]
TUG, cogTUG	Sit-to-Stand	Jerk sit-to-stand B [g/s]
TUG, cogTUG	Stand-to-Sit	Jerk stand-to-sit A [g/s]
TUG, cogTUG	Stand-to-Sit	Jerk stand-to-sit B [g/s]
TUG, cogTUG	Sit-to-Stand	Range sit-to-stand A [g]
TUG, cogTUG	Sit-to-Stand	Range sit-to-stand B [g]
TUG, cogTUG	Stand-to-Sit	Range stand-to-sit A [g]
TUG, cogTUG	Stand-to-Sit	Range stand-to-sit B [g]
TUG, cogTUG	Sit-to-Stand	Median [g]
TUG, cogTUG	Stand-to-Sit	Median [g]
TUG, cogTUG	Sit-to-Stand	Standard deviation [g]
TUG, cogTUG	Stand-to-Sit	Standard deviation [g]
TUG, cogTUG	Sit-to-Stand	Minimum amplitude [deg/sec]
TUG, cogTUG	Stand-to-Sit	Minimum amplitude [deg/sec]
TUG, cogTUG	Sit-to-Stand	Duration pitch [s]
TUG, cogTUG	Sit-to-Stand	Range pitch [deg/s]
TUG, cogTUG	Sit-to-Stand	Jerk pitch [deg/s^2]

TUG, cogTUG	Stand-to-Sit	Duration pitch [s]
TUG, cogTUG	Stand-to-Sit	Range pitch [deg/s]
TUG, cogTUG	Stand-to-Sit	Jerk pitch [deg/s^2]
TUG, cogTUG	Sit-to-Stand	Duration integrated pitch [s]
TUG, cogTUG	Sit-to-Stand	Range of integrated pitch [deg]
TUG, cogTUG	Sit-to-Stand	Minimum amplitude of integrated pitch [deg]
TUG, cogTUG	Stand-to-Sit	Duration integrated pitch [s]
TUG, cogTUG	Stand-to-Sit	Range of integrated pitch [deg]
TUG, cogTUG	Stand-to-Sit	Minimum amplitude of integrated pitch [deg]
TUG, cogTUG	Sit-to-Stand	Slope of the pitch [deg/s^2]
TUG, cogTUG	Stand-to-Sit	Slope of the pitch [deg/s^2]
TUG, cogTUG	Sit-to-Stand	Mean error between the pitch and its linear fit [deg/s^2]
TUG, cogTUG	Stand-to-Sit	Mean error between the pitch and its linear fit [deg/s^2]
TUG, cogTUG	Turn 1,2	Amplitude-yaw [deg/s]
TUG, cogTUG	Turn 1,2	Duration [s]
TUG, cogTUG	Turn 1,2	Dominant frequency [hz]
TUG, cogTUG	Turn 1,2	Number of steps
16-foot walk	Turn	Duration [s]
16-foot walk	Turn	Dominant frequency [hz]
16-foot walk	Turn	Number of steps
16-foot walk	Walking 1,2	Duration [s]
16-foot walk	Walking 1,2	Number of steps
16-foot walk	Walking 1,2	Step duration [s]
16-foot walk	Walking 1,2	Stride duration [s]
16-foot walk	Walking 1,2	Step duration/stride duration
16-foot walk	Walking 1,2	Frequency (7 s) [hz]
16-foot walk	Walking 1,2	Amplitude (7 s) [psd]
16-foot walk	Walking 1,2	Width (7 s) [hz]
16-foot walk	Walking 1,2	Slope (7 s) [psd/hz]
16-foot walk	Walking 1,2	Number of steps
16-foot walk	Walking 1,2	Step regularity [g^2]
16-foot walk	Walking 1,2	Stride regularity [g^2]
16-foot walk	Walking 1,2	Step symmetry

Table S2. Intraclass correlation coefficient (ICC, 95% CI) of sensor-based total duration of each mobility task. CI: confidence interval.

Task	Controls	Mild PD	Moderate PD	Severe PD
TUG	0.87 (0.63-0.94)	0.82 (0.72-0.87)	0.87 (0.76-0.93)	0.95 (0.87-0.98)
cogTUG	0.84 (0.50-0.94)	0.85 (0.76-0.90)	0.75 (0.61-0.85)	0.88 (0.69-0.95)
16-foot walk	0.71 (-0.03-0.90)	0.63 (0.06-0.83)	0.62 (0.15-0.82)	0.77 (0.45-0.91)

Table S3. Variance of sensor-based task duration (s^2).

Task	Controls		Mild PD		Moderate PD		Severe PD	
	Trial 1	Trial2	Trial 1	Trial2	Trial 1	Trial2	Trial 1	Trial2
TUG	6.46	4.64	11.34	9.32	26.44	18.71	327.84	277.06
cogTUG	13.05	11.96	46.28	39.65	56.97	54.95	434.26	371.90
16-foot walk	1.82	2.62	3.00	5.52	5.90	9.34	36.24	109.10

Table S4. Values of assessments measures.

Assessment	Measure	Trial 1 mean ± SD				Trial 2 mean ± SD			
		Controls	mild PD	moderate PD	severe PD	Controls	mild PD	moderate PD	severe PD
TUG	Total Duration [s]	12.05 ± 2.54	14.48 ± 3.37	17.1 ± 5.14	29.46 ± 18.09	11.34 ± 2.15	13.77 ± 3.01	16.08 ± 4.33	28.38 ± 16.48
	[Turn 2] Amplitude-Yaw [deg/s]	199.86 ± 42.16	167.23 ± 46.61	138.74 ± 53.79	93.36 ± 35.49	208.19 ± 40.37	170.7 ± 42.7	146.4 ± 47.7	89.46 ± 34.86
	[Sit-to-Stand] Jerk [g/s]	-2.54 ± 0.94	-3.91 ± 11.2	-2.92 ± 9.25	-0.56 ± 0.76	-3.44 ± 3.05	-2.76 ± 5.98	-1.52 ± 1.37	-2.53 ± 6.99
	[Sit-to-Stand] Range pitch [deg/s]	361.84 ± 128.48	280.14 ± 140.56	210.5 ± 88.78	141.85 ± 110.9	360.8 ± 118.4	262.3 ± 130.3	214.3 ± 113.8	182.9 ± 130.3
	[Sit-to-Stand] Jerk Sit-to-Stand A [g/s]	-2.6 ± 2.36	-2.99 ± 10.11	-2.85 ± 8.86	-0.71 ± 0.91	-3.7 ± 3.63	-3.01 ± 6.74	-1.6 ± 1.43	-2.16 ± 5.52
	[Sit-to-Stand] Range [g]	1.48 ± 0.35	1.28 ± 0.48	1.02 ± 0.31	0.82 ± 0.32	1.52 ± 0.35	1.27 ± 0.46	1.07 ± 0.35	0.98 ± 0.5
	[Sit-to-Stand] Mean error between the pitch and it's linear fit [deg/s^2]	37.57 ± 21.99	24.07 ± 19.51	17.4 ± 15.16	13.2 ± 13.34	36.66 ± 20.88	20.59 ± 18.9	16.67 ± 18.63	15.14 ± 11.29
	[Stand-to-Sit] Mean error between the pitch and it's linear fit [deg/s^2]	14.71 ± 15.48	18.44 ± 11.75	15.51 ± 8.82	10.72 ± 6.16	13.48 ± 10.61	14.85 ± 11.58	12.82 ± 9.62	10.87 ± 5.86
	[Stand-to-Sit] Jerk pitch [deg/s^2]	205.33 ± 145.88	368.49 ± 2607.44	314.36 ± 1128.7	58.67 ± 46.01	201.83 ± 126.64	109.4 ± 609.3	374.1 ± 1921.4	55.82 ± 43.87
	[Sit-to-Stand] Maximum amplitude [deg/s]	65.71 ± 35.45	65.11 ± 51.54	58.3 ± 31.99	27.47 ± 20.21	66.82 ± 38.41	62.69 ± 41.81	53.6 ± 30.73	50.54 ± 36.72
	[Stand-to-Sit] Range [g]	1.83 ± 0.73	1.44 ± 0.51	1.3 ± 0.44	1.36 ± 0.56	1.75 ± 0.58	1.42 ± 0.53	1.36 ± 0.52	1.01 ± 0.36
	[Stand-to-Sit] Range Stand-to-Sit B [g]	1.41 ± 0.74	1.02 ± 0.57	0.94 ± 0.51	1.08 ± 0.63	1.25 ± 0.65	1 ± 0.58	1 ± 0.59	0.67 ± 0.4
cogTUG	Total Duration [s]	15 ± 3.61	19.8 ± 6.8	24.37 ± 7.55	39.98 ± 20.84	13.71 ± 3.46	18.33 ± 6.3	22.75 ± 7.41	36.1 ± 19.28
	[Sit-to-Stand] Jerk [g/s]	-2.39 ± 1.14	-1.45 ± 1.68	-0.95 ± 0.74	-0.37 ± 0.74	-2.59 ± 1.65	-1.4 ± 0.86	-1.04 ± 1.04	-0.41 ± 0.75
	[Sit-to-Stand] Slope of the pitch [deg/s^2]	410.67 ± 167.04	317.21 ± 279.54	238.99 ± 207.02	152.75 ± 134.4	424.02 ± 217.61	302.34 ± 207.53	221.39 ± 181.47	155.21 ± 94.84
	[Stand-to-Sit] Jerk Pitch [deg/s^2]	240.05 ± 160.25	156.17 ± 350.04	107.88 ± 78.21	69.71 ± 172.68	246.95 ± 168.12	158.7 ± 129.8	103.48 ± 92.57	290.1 ± 989.16
	[Sit-to-Stand] Jerk Sit-to-Stand A [g/s]	-2.03 ± 1.54	-0.9 ± 1.6	-0.8 ± 0.84	-0.36 ± 0.49	-2.3 ± 1.73	-0.88 ± 1.07	-0.94 ± 1.21	-0.06 ± 1.54
	[Turn 2] Amplitude-Yaw [deg/s]	183.92 ± 48.01	143.98 ± 43.77	116.08 ± 39.71	94.58 ± 75.08	198.91 ± 58.91	149.17 ± 48.93	126.97 ± 49.77	95.28 ± 60.64
	[Sit-to-Stand] Range [g]	1.43 ± 0.4	1.17 ± 0.45	0.98 ± 0.24	0.93 ± 0.52	1.48 ± 0.36	1.12 ± 0.39	0.97 ± 0.25	0.88 ± 0.38
	[Stand-to-Sit] Range [g]	1.94 ± 0.72	1.33 ± 0.49	1.48 ± 0.62	1.25 ± 1.05	1.84 ± 0.69	1.4 ± 0.6	1.41 ± 0.55	1.07 ± 0.38
	[Sit-to-Stand] Standard deviation [g]	0.39 ± 0.09	0.31 ± 0.11	0.28 ± 0.07	0.22 ± 0.09	0.4 ± 0.09	0.3 ± 0.09	0.28 ± 0.08	0.24 ± 0.09
	[Stand-to-Sit] Jerk Stand-to-Sit B [g/s]	1.31 ± 0.84	1 ± 2.7	1.02 ± 1.23	0.47 ± 0.93	1.48 ± 0.86	1.27 ± 1.88	1.16 ± 0.96	0.54 ± 0.69
	[Turn 1] Amplitude-Yaw [deg/s]	181.3 ± 46.29	148.01 ± 41.12	130.09 ± 38.96	106.79 ± 75.12	180.36 ± 44.81	152.87 ± 39.82	128.77 ± 36.15	103.44 ± 53.24
	[Sit-to-Stand] Range Sit-to-Stand A [g]	0.78 ± 0.5	0.57 ± 0.37	0.54 ± 0.29	0.51 ± 0.44	0.88 ± 0.41	0.56 ± 0.41	0.54 ± 0.31	0.5 ± 0.46
16-foot walk	Total Duration [s]	7.35 ± 1.35	7.79 ± 1.73	9.51 ± 2.43	14.37 ± 6.02	8.36 ± 1.62	9.2 ± 2.35	11.23 ± 3.06	17.06 ± 10.45
	[Turn] Duration [s]	1.56 ± 0.36	1.7 ± 0.48	2.06 ± 0.68	2.5 ± 0.82	1.55 ± 0.38	1.72 ± 0.53	2.18 ± 0.75	2.44 ± 0.8
	[Walking 1] Stride Regularity [g^2]	0.65 ± 0.17	0.64 ± 0.19	0.66 ± 0.32	0.58 ± 0.22	0.77 ± 0.18	0.66 ± 0.26	0.63 ± 0.22	0.61 ± 0.19
	[Walking 1] Duration- Walk 1 [s]	2.96 ± 0.62	3.07 ± 0.71	3.7 ± 1.04	4.98 ± 2.61	2.89 ± 0.92	3.29 ± 1.12	4.27 ± 1.51	7.74 ± 8.7
	[Walking 1] Step Regularity [g^2]	0.73 ± 0.18	0.65 ± 0.23	0.52 ± 0.29	0.39 ± 0.31	0.69 ± 0.27	0.6 ± 0.26	0.5 ± 0.24	0.34 ± 0.24
	[Turn] Number of steps to complete turn	1.8 ± 1.32	1.53 ± 1.75	2.45 ± 2.14	0.83 ± 1.19	1.41 ± 1.26	1.92 ± 2.15	1.95 ± 2.22	1.07 ± 0.97
	[Walking 2] Step duration [s]	0.62 ± 0.1	0.59 ± 0.11	0.61 ± 0.09	0.71 ± 0.28	0.62 ± 0.17	0.6 ± 0.14	0.62 ± 0.12	0.68 ± 0.24
	[Walking 1] Number of steps to complete walk	5.1 ± 1.2	5.38 ± 1.36	6.25 ± 1.93	7.41 ± 2.32	4.74 ± 1.14	5.48 ± 1.88	6.52 ± 2.38	9.35 ± 6.44
	[Walking 2] Step Regularity [g^2]	0.69 ± 0.25	0.65 ± 0.26	0.49 ± 0.29	0.32 ± 0.22	0.64 ± 0.15	0.59 ± 0.2	0.46 ± 0.24	0.39 ± 0.25
	[Walking 2] Stride duration [s]	1.24 ± 0.23	1.17 ± 0.18	1.23 ± 0.16	1.47 ± 0.68	1.24 ± 0.34	1.18 ± 0.22	1.23 ± 0.22	1.34 ± 0.44
	[Turn] Dominant frequency [Hz]	1.12 ± 0.8	0.91 ± 1.13	1.31 ± 1.28	0.34 ± 0.5	0.87 ± 0.69	1.13 ± 1.24	0.97 ± 1.12	0.49 ± 0.47
	[Walking 2] Stride Regularity [g^2]	0.72 ± 0.2	0.73 ± 0.31	0.58 ± 0.27	0.4 ± 0.28	0.59 ± 0.16	0.59 ± 0.19	0.5 ± 0.17	0.52 ± 0.23

Table S5. Reliability of each of TUG, cogTUG, and 16-foot walk assessments. ICC intraclass correlation coefficient, CI confidence interval, SEM standard error of measurement agreement, MDC minimal detectable change.

Assessment	Measure	ICC (95%CI)				SEM				MDC			
		Controls	mild PD	moderate PD	severe PD	Controls	mild PD	moderate PD	severe PD	Controls	mild PD	moderate PD	severe PD
TUG	Total Duration [s]	0.87 (0.63-0.94)	0.82 (0.72-0.87)	0.87 (0.76-0.93)	0.95 (0.87-0.98)	0.85	1.38	1.7	3.78	2.35	3.82	4.7	10.47
	[Turn 2] Amplitude-Yaw [deg/s]	0.78 (0.64-0.87)	0.71 (0.63-0.77)	0.73 (0.59-0.83)	0.74 (0.41-0.90)	19.24	24.11	26.35	17.73	53.34	66.83	73.05	49.14
	[Sit-to-Stand] Jerk [g/s]	0.00 (-0.26-0.26)	0.38 (0.24-0.49)	0.02 (-0.23-0.27)	0.03 (-0.44-0.50)	2.29	7.1	6.54	4.91	6.35	19.68	18.12	13.61
	[Sit-to-Stand] Range pitch [deg/s]	0.54 (0.32-0.71)	0.59 (0.49-0.68)	0.75 (0.62-0.84)	0.66 (0.27-0.86)	83.13	86.92	50.48	70.64	230.41	240.92	139.92	195.8
	[Sit-to-Stand] Jerk Sit-to-Stand A [g/s]	0.27 (0.01-0.50)	0.33 (0.19-0.45)	0.01 (-0.24-0.26)	0.16 (-0.34-0.59)	2.65	7.02	6.31	3.64	7.34	19.47	17.48	10.09
	[Sit-to-Stand] Range [g]	0.56 (0.34-0.72)	0.56 (0.45-0.65)	0.58 (0.39-0.73)	0.60 (0.19-0.84)	0.23	0.31	0.21	0.27	0.64	0.86	0.59	0.74
	[Sit-to-Stand] Mean error between the pitch and it's linear fit [deg/s^2]	0.68 (0.50-0.80)	0.49 (0.37-0.59)	0.73 (0.59-0.83)	0.40 (-0.09-0.74)	12.06	13.76	8.8	9.47	33.43	38.14	24.4	26.26
	[Stand-to-Sit] Mean error between the pitch and it's linear fit [deg/s^2]	0.38 (0.12-0.59)	0.44 (0.30-0.55)	0.47 (0.25-0.64)	0.09 (-0.41-0.55)	10.42	8.85	6.77	5.64	28.88	24.54	18.76	15.63
	[Stand-to-Sit] Jerk pitch [deg/s^2]	0.70 (0.53-0.82)	0.01 (-0.13-0.16)	0.84 (0.74-0.90)	0.49 (0.02-0.78)	74.1	1881.68	634.86	31.64	205.4	5215.75	1759.74	87.71
	[Sit-to-Stand] Maximum amplitude [deg/s]	0.90 (0.82-0.94)	0.30 (0.16-0.43)	0.00 (-0.25-0.25)	0.36 (-0.07-0.71)	11.86	39.25	31.32	25.06	32.88	108.78	86.83	69.47
	[Stand-to-Sit] Range [g]	0.33 (0.06-0.55)	0.61 (0.51-0.69)	0.70 (0.54-0.81)	0.26 (-0.14-0.64)	0.54	0.33	0.26	0.42	1.49	0.9	0.73	1.18
	[Stand-to-Sit] Range Stand-to-Sit B [g]	0.31 (0.04-0.54)	0.57 (0.46-0.66)	0.72 (0.58-0.83)	0.15 (-0.22-0.55)	0.58	0.38	0.29	0.52	1.6	1.05	0.8	1.43
cogTUG	Total Duration [s]	0.84 (0.49-0.94)	0.85 (0.76-0.90)	0.75 (0.61-0.85)	0.88 (0.69-0.95)	1.42	2.53	3.72	7	3.93	7.02	10.31	19.41
	[Sit-to-Stand] Jerk [g/s]	0.31 (0.04-0.54)	0.18 (0.03-0.31)	0.53 (0.32-0.69)	0.86 (0.65-0.94)	1.18	1.21	0.62	0.28	3.26	3.36	1.72	0.77
	[Sit-to-Stand] Slope of the pitch [deg/s^2]	0.57 (0.35-0.73)	0.51 (0.40-0.61)	0.67 (0.50-0.79)	0.74 (0.42-0.90)	126.65	171.32	112.17	58.65	351.05	474.87	310.91	162.56
	[Stand-to-Sit] Jerk pitch [deg/s^2]	0.81 (0.69-0.89)	0.00 (-0.14-0.14)	0.66 (0.49-0.78)	0.00 (-0.47-0.47)	71.28	263.63	50.05	708.07	197.56	730.74	138.74	1962.66
	[Sit-to-Stand] Jerk Sit-to-Stand A [g/s]	0.07 (-0.21-0.34)	0.15 (0.00-0.29)	0.66 (0.50-0.78)	0.34 (-0.14-0.70)	1.58	1.26	0.6	0.92	4.38	3.48	1.67	2.55
	[Turn 2] Amplitude-Yaw [deg/s]	0.67 (0.47-0.80)	0.69 (0.61-0.76)	0.72 (0.56-0.83)	0.86 (0.66-0.95)	31.14	25.73	23.83	25.18	86.32	71.31	66.05	69.8
	[Sit-to-Stand] Range [g]	0.39 (0.13-0.60)	0.67 (0.58-0.74)	0.44 (0.21-0.62)	0.79 (0.52-0.92)	0.3	0.24	0.18	0.21	0.83	0.67	0.5	0.57
	[Stand-to-Sit] Range [g]	0.68 (0.50-0.81)	0.56 (0.46-0.66)	0.71 (0.56-0.82)	0.35 (-0.13-0.70)	0.4	0.36	0.31	0.63	1.1	1	0.87	1.74
	[Sit-to-Stand] Standard deviation [g]	0.46 (0.22-0.66)	0.57 (0.47-0.66)	0.56 (0.36-0.71)	0.79 (0.52-0.92)	0.06	0.06	0.05	0.04	0.17	0.18	0.14	0.11
	[Stand-to-Sit] Jerk Stand-to-Sit B [g/s]	0.18 (-0.10-0.43)	0.56 (0.45-0.65)	0.63 (0.45-0.76)	0.34 (-0.14-0.70)	0.77	1.55	0.67	0.65	2.14	4.29	1.85	1.81
	[Turn 1] Amplitude-Yaw [deg/s]	0.90 (0.83-0.94)	0.77 (0.70-0.82)	0.81 (0.70-0.88)	0.89 (0.74-0.96)	14.55	19.56	16.4	20.96	40.32	54.22	45.46	58.09
	[Sit-to-Stand] Range Sit-to-Stand A [g]	0.21 (-0.06-0.46)	0.31 (0.17-0.43)	0.34 (0.10-0.55)	0.00 (-0.47-0.47)	0.4	0.33	0.24	0.44	1.12	0.9	0.67	1.23
16-foot walk	Total Duration [s]	0.71 (-0.03-0.90)	0.63 (0.06-0.83)	0.62 (0.15-0.82)	0.77 (0.45-0.91)	0.84	1.32	1.78	4.09	2.33	3.66	4.92	11.34
	[Turn] Duration [s]	0.57 (0.35-0.73)	0.73 (0.65-0.79)	0.8 (0.68-0.88)	0.65 (0.26-0.85)	0.24	0.26	0.32	0.48	0.67	0.73	0.89	1.32
	[Walking 1] Stride Regularity [g^2]	0.00 (-0.21-0.24)	0.22 (0.08-0.35)	0.00 (-0.25-0.25)	0.12 (-0.37-0.55)	0.18	0.2	0.27	0.19	0.5	0.55	0.76	0.53
	[Walking 1] Duration- Walk 1 [s]	0.58 (0.36-0.74)	0.5 (0.38-0.60)	0.32 (0.09-0.53)	0.28 (-0.18-0.65)	0.51	0.67	1.09	5.5	1.41	1.86	3.01	15.25
	[Walking 1] Step Regularity [g^2]	0.08 (-0.20-0.34)	0.34 (0.21-0.46)	0.38 (0.14-0.57)	0.45 (-0.01-0.76)	0.22	0.2	0.21	0.2	0.61	0.56	0.59	0.56
	[Turn] Number of steps to complete turn	0.40 (0.15-0.61)	0.06 (-0.08-0.20)	0.39 (0.16-0.59)	0.34 (-0.14-0.70)	1	1.91	1.7	0.88	2.78	5.28	4.72	2.43
	[Walking 2] Step duration [s]	0.52 (0.29-0.70)	0.36 (0.22-0.48)	0.54 (0.33-0.70)	0.37 (-0.11-0.71)	0.1	0.1	0.07	0.2	0.27	0.28	0.19	0.56
	[Walking 1] Number of steps to complete walk	0.26 (0.00-0.05)	0.39 (0.26-0.51)	0.41 (0.18-0.60)	0.03 (-0.43-0.48)	1.01	1.28	1.66	4.8	2.8	3.55	4.6	13.31
	[Walking 2] Step Regularity [g^2]	0.31 (0.05-0.54)	0.42 (0.29-0.53)	0.42 (0.19-0.61)	0.50 (0.06-0.78)	0.17	0.18	0.2	0.16	0.48	0.5	0.56	0.45
	[Walking 2] Stride duration [s]	0.54 (0.31-0.71)	0.5 (0.38-0.60)	0.47 (0.25-0.65)	0.22 (-0.27-0.62)	0.19	0.14	0.14	0.5	0.54	0.4	0.39	1.39
	[Turn] Dominant frequency [Hz]	0.35 (0.10-0.57)	0.02 (-0.13-0.16)	0.23 (-0.02-0.45)	0.17 (-0.32-0.59)	0.61	1.18	1.06	0.44	1.68	3.27	2.95	1.22
	[Walking 2] Stride Regularity [g^2]	0.10 (-0.13-0.33)	0.19 (0.05-0.33)	0.30 (0.06-0.51)	0.53 (0.10-0.80)	0.18	0.24	0.19	0.18	0.5	0.67	0.52	0.5

Table S6. Median of intraclass correlation coefficient (median, 95% confidence interval (CI)) across the quantitative measures.

Task	Controls	Mild PD	Moderate PD	Severe PD
TUG	0.50 (0.32-0.67)	0.45 (0.32-0.58)	0.51 (0.28-0.73)	0.36 (0.29-0.52)
cogTUG	0.48 (0.29-0.66)	0.45 (0.28-0.62)	0.61 (0.52-0.70)	0.54 (0.31-0.75)
16-foot walk	0.35 (0.23-0.48)	0.35 (0.23-0.46)	0.39 (0.28-0.49)	0.34 (0.23-0.44)

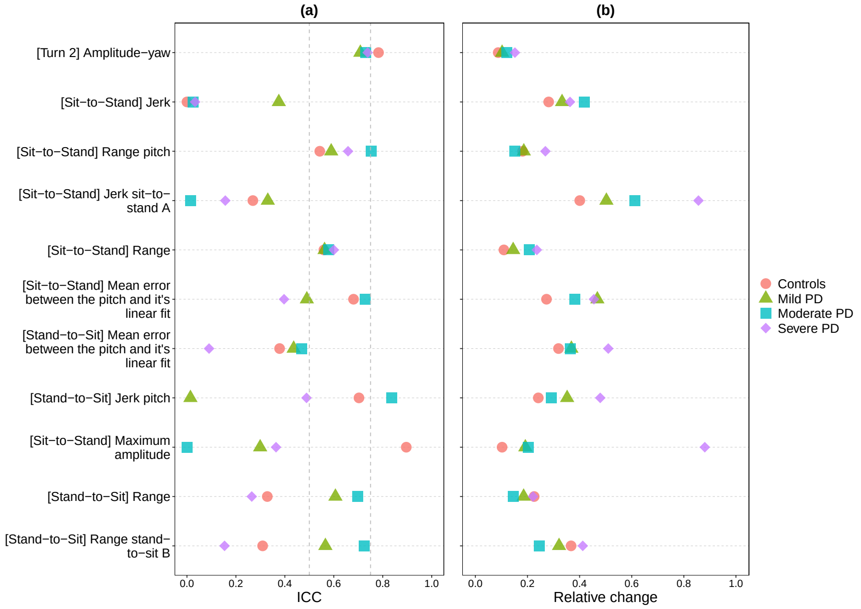

Figure S1. TUG intraclass correlation coefficient (ICC) **(a)** and median of the relative change **(b)** of each measure. Vertical dashed lines represent thresholds for moderate and good reliability.

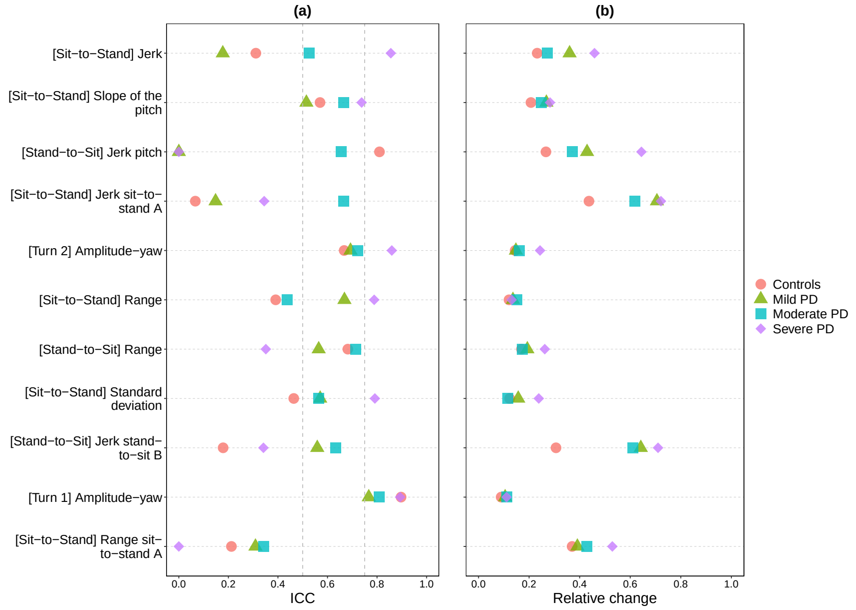

Figure S2. cogTUG intraclass correlation coefficient (ICC) **(a)** and median of the relative change **(b)** of each measure. Vertical dashed lines represent thresholds for moderate and good reliability.

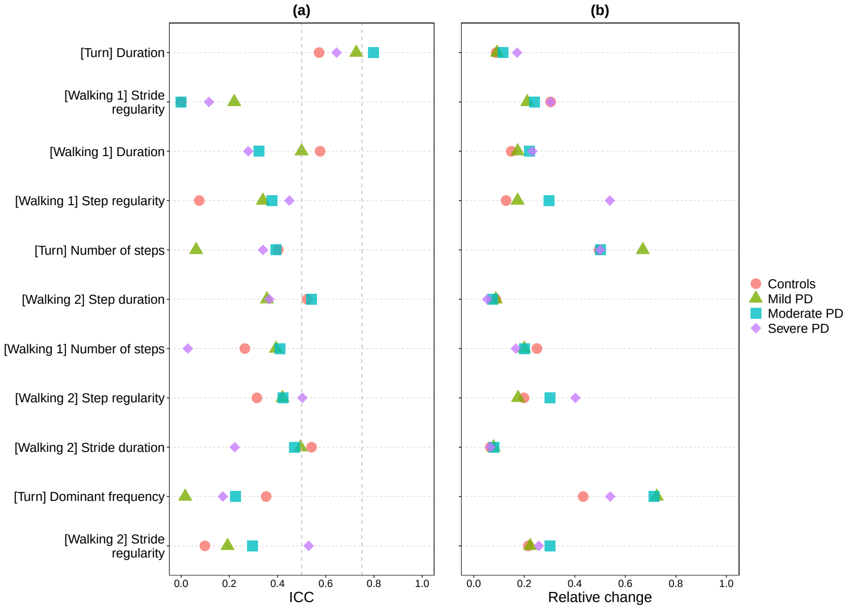

Figure S3. 16-foot walk intraclass correlation coefficient (ICC) **(a)** and median of the relative change **(b)** of each measure. Vertical dashed lines represent thresholds for moderate and good reliability.

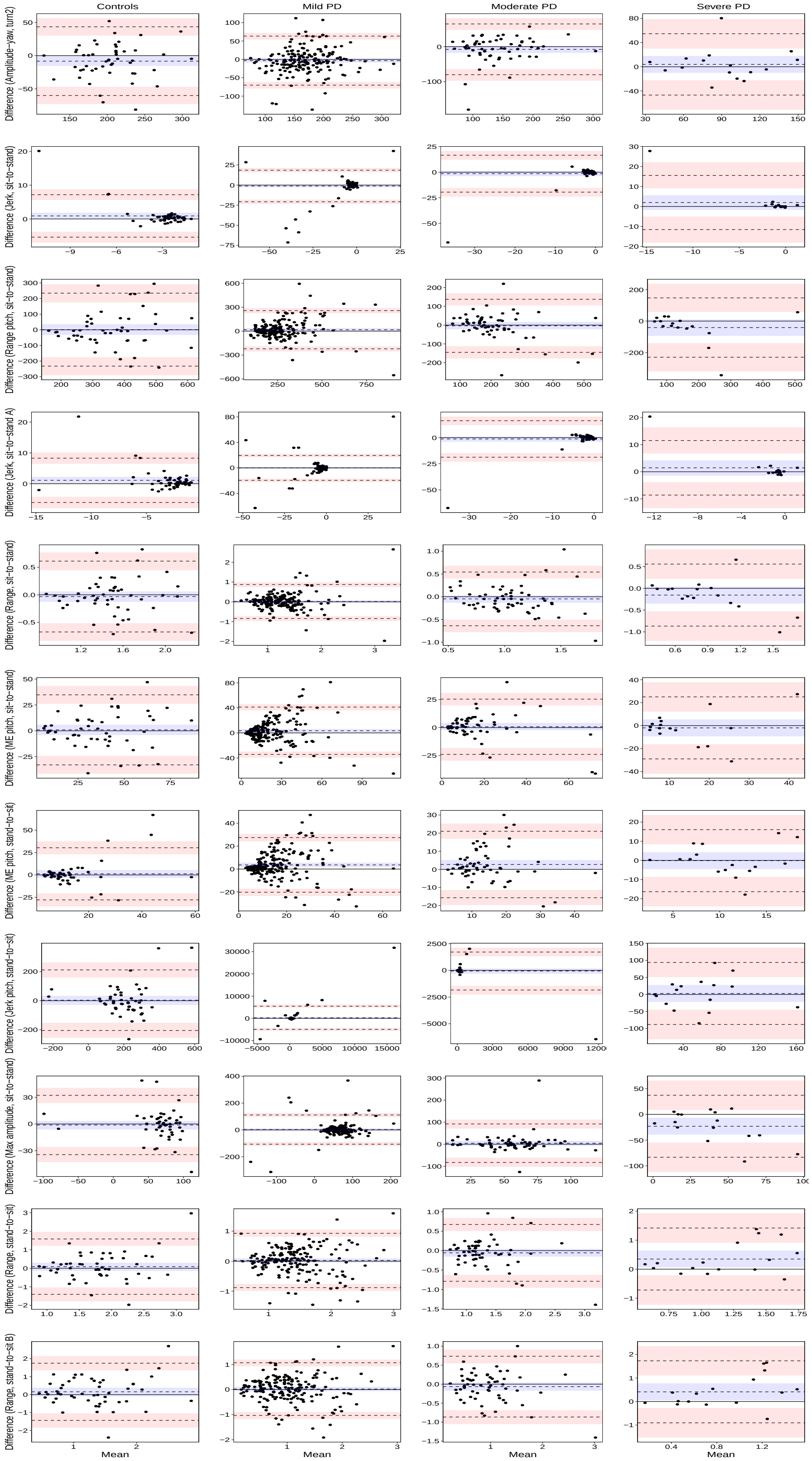

Figure S4. Bland-Altman plots of each measure for the TUG task. The dashed lines represent the mean of the differences and the 95% limits of agreement (LoA), defined as mean ± 1.96 SD. The blue and red shaded areas are the confidence intervals of the mean and LoA respectively.

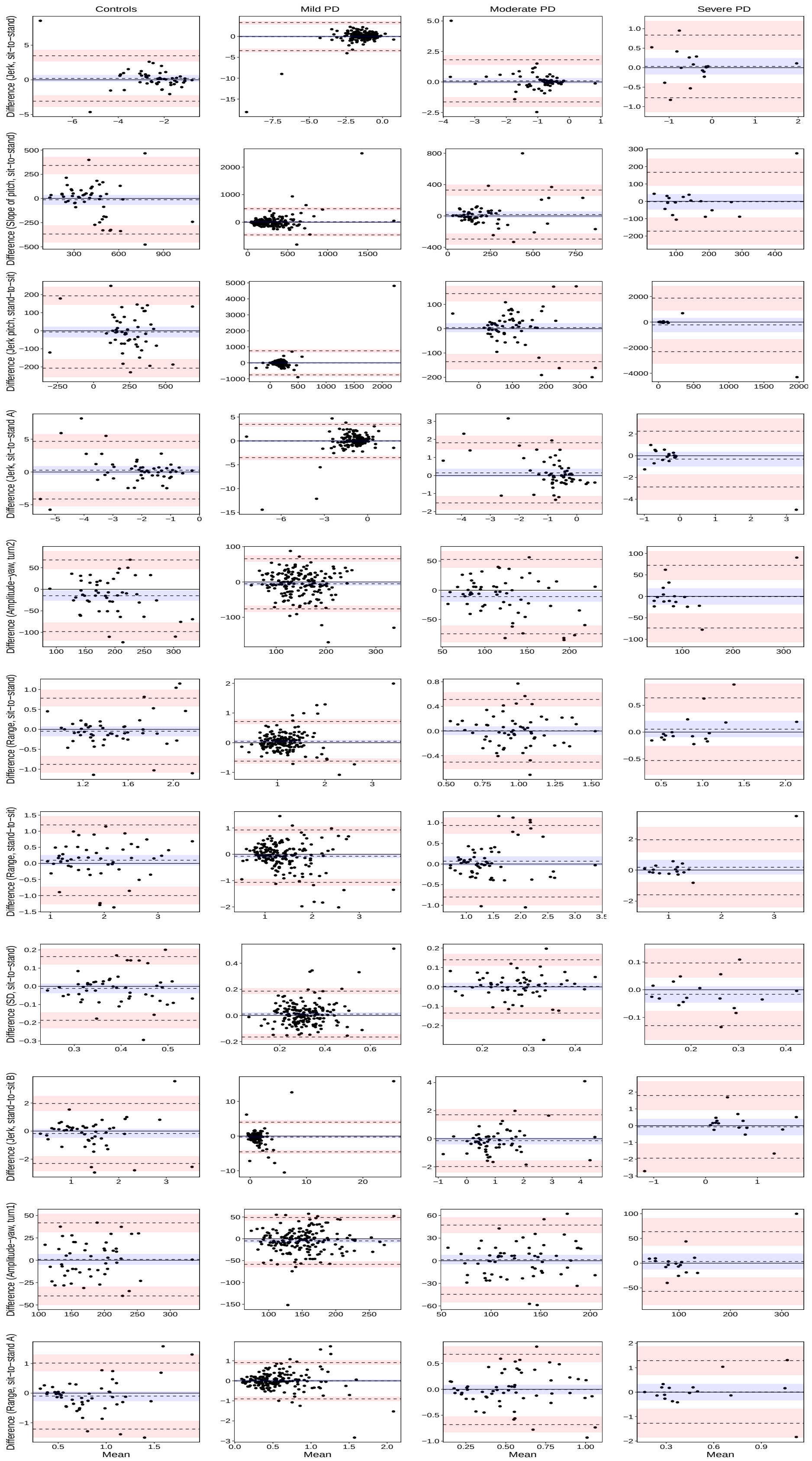

Figure S5. Bland-Altman plots of each measure for the cogTUG task. The dashed lines represent the mean of the differences and the 95% limits of agreement (LoA), defined as mean ± 1.96 SD. The blue and red shaded areas are the confidence intervals of the mean and LoA respectively.

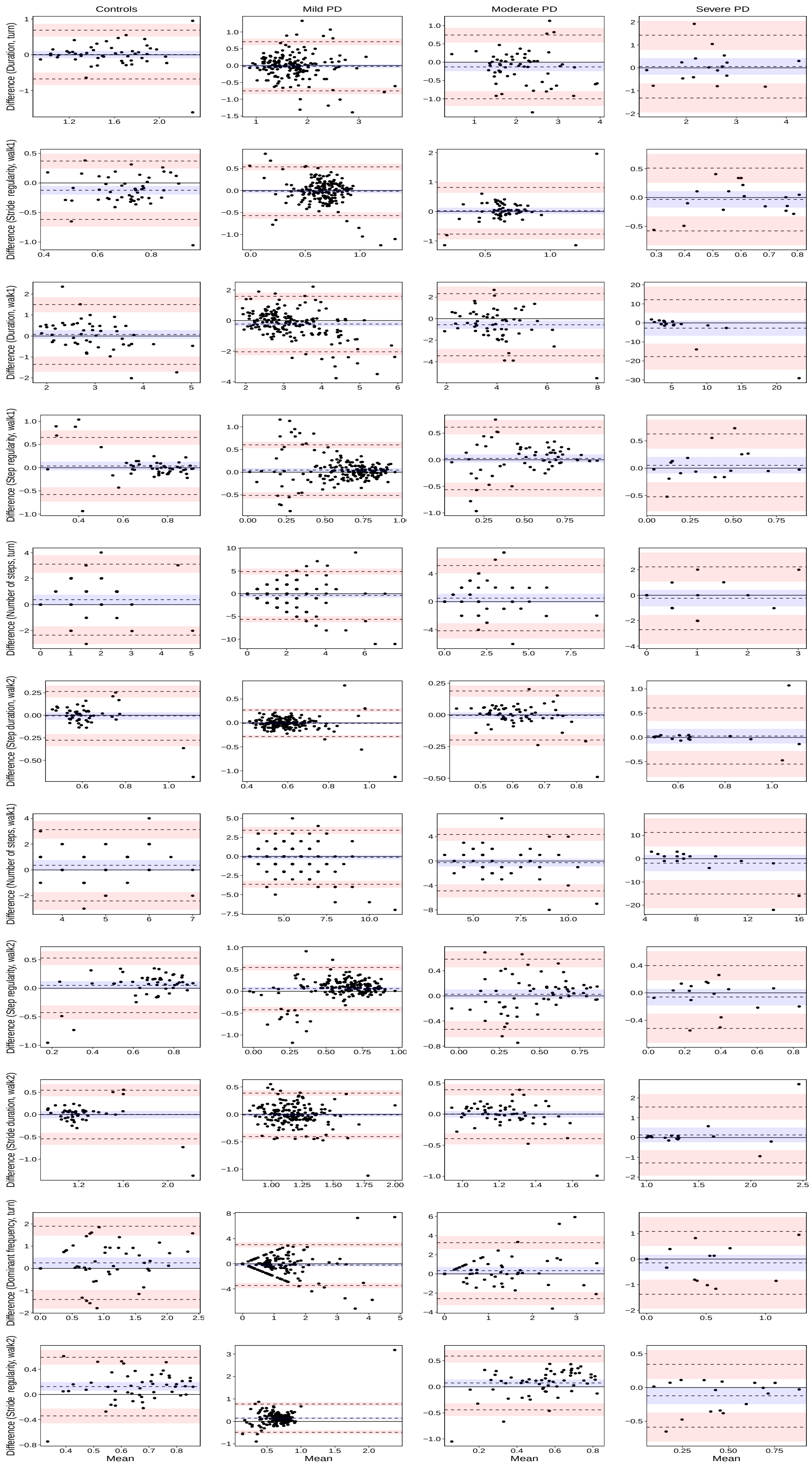

Figure S6. Bland-Altman plots of each measure for the 16-foot walk task. The dashed lines represent the mean of the differences and the 95% limits of agreement (LoA), defined as mean ± 1.96 SD. The blue and red shaded areas are the confidence intervals of the mean and LoA respectively.

Supplementary Files

The supplementary material available at... contains the following compressed files:

1. **data**: folder with quantitative measures derived from two trials of TUG, cogTUG, and 16-foot walk.

2. **code_notebook**: notebook with the code used to generate the results provided in the manuscript. To view the notebook open the file `index.html` in a web browser or open the file `notebook.pdf`.

3. **rdata**: folder with intermediate R objects.

   `tug_cogtug_durations.RData` saves the total duration and the subtask duration of all participants for both trials of TUG and cogTUG.

   `16ft_durations.RData` saves the total duration and the subtask duration of all participants for 16-foot walk.

   `ids.RData`: saves the ids of the mild, moderate, and severe PD participants.

   `HY_early_phys_dfs.RData`: saves the quantitative measures of the mild PD participants.

   `HY_mild_phys_dfs.RData`: saves the quantitative measures of the moderate PD participants.

   `HY_severe_phys_dfs.RData`: saves the quantitative measures of the severe PD participants.

   `HY_control_early_tug_phys.RData`: save a data frame with rows corresponding to mild PD participants and controls and columns corresponding to the quantitative measures derived from both TUG trials and selected by the feature reduction technique.

   `HY_control_early_cogtug_phys.RData`: save a data frame with rows corresponding to mild PD participants and controls and columns corresponding to the quantitative measures derived from both cogTUG trials and selected by the feature reduction technique.

   `HY_control_early_trial_32ft_phys.RData`: save a data frame with rows corresponding to mild PD participants and controls and columns corresponding to the quantitative measures derived from both 16-foot walk trials and selected by the feature reduction technique.

   `HY_control_mild_tug_phys.RData`: save a data frame with rows corresponding to moderate PD participants and controls and columns corresponding to the quantitative measures derived from both TUG trials and selected by the feature reduction technique.

   `HY_control_mild_cogtug_phys.RData`: save a data frame with rows corresponding to moderate PD participants and controls and columns corresponding to the quantitative measures derived from both cogTUG trials and selected by the feature reduction technique.

   `HY_control_mild_trial_32ft_phys.RData`: save a data frame with rows corresponding to moderate PD participants and controls and columns corresponding to the quantitative measures derived from both 16-foot walk trials and selected by the feature reduction technique.

   `HY_control_severe_tug_phys.RData`: save a data frame with rows corresponding to severe PD participants and controls and columns corresponding to the quantitative measures derived from both TUG trials and selected by the feature reduction technique.

   `HY_control_severe_cogtug_phys.RData`: save a data frame with rows corresponding to severe PD participants and controls and columns corresponding to the quantitative measures derived from both cogTUG trials and selected by the feature reduction technique.

   `HY_control_severe_trial_32ft_phys.RData`: save a data frame with rows corresponding to severe PD participants and controls and columns corresponding to the quantitative measures derived from both 16-foot walk trials and selected by the feature reduction technique.

   `HY_early_HC_phys_only_splits.RData` saves the training and test splits for the five repeats and five-fold cross-validation framework used to build classifiers distinguishing mild PD participants and controls.

   `HY_mild_HC_phys_only_splits.RData` saves the training and test splits for the five repeats and five-fold cross-validation framework used to build classifiers distinguishing moderate PD participants and controls.

   `HY_severe_HC_phys_only_splits.RData` saves the training and test splits for the five repeats and five-fold cross-validation framework used to build classifiers distinguishing severe PD participants and controls.

4. **files**: For each mobility task, trial, and group of PD participants, there are five `.csv` files representing the random forest predictions of the five repeats.

5. **measures_ICC**: Folder with ICC values for all quantitative measures of each mobility task.

6. `quantitative_measures.csv`: a file with all sensor-derived quantitative measures included in the analysis of the manuscript, along with a brief description of each.

7. **README**: file with detailed instructions on how to set up and run the code, as well as any dependencies or requirements.

References
